# Contemporary Cardiac Surgery—Towards Granular Precision in Surgical Care

**DOI:** 10.3390/jcdd13070296

**Published:** 2026-06-26

**Authors:** Jae Hwan Choi

**Affiliations:** Department of Surgery, University of South Florida, Tampa, FL 33612, USA; choijaehwan1@gmail.com

The recent advancement in cardiovascular surgery continues to move at a remarkable pace, driven not only by developments in surgical techniques, but also through collaborations from multiple disciplines to optimize patient care [1]. This collective effort within the field, with the integration of clinical data which previously helped establish phenotypes for cardiac pathology, now lends itself to newer concept of “precision medicine” which considers individual factors in the clinical decision-making process [2]. This Special Issue of The *Journal of Cardiovascular Development and Disease* seeks to highlight up-to-date reviews and original investigations that may help identify factors to improve our clinical acumen in this highly sophisticated field.

A recurring theme throughout this Special Issue is the increasing emphasis on refinement within established interventions, particularly through improved perioperative measurements and planning for safe cardiac interventions. Recent advances in imaging techniques now allow better procedural planning and patient selections, especially in technically challenging scenarios. In this context, Napoli et al. showcase novel reports of transcatheter aortic valve replacement in raphe-type bicuspid aortic valve patients with severe valvular stenosis, utilizing the LIRA method with state-of-the-art valvular prosthesis for more accurate sizing in this difficult patient population with higher risks of paravalvular leaks [3]. Complementing this concept of image-guided interventions, Piotrowski et al. report the importance of topographic relations between the tricuspid valve and right coronary artery, identifying the posterior aspect of the tricuspid annulus as the most likely site for iatrogenic coronary injury. Meanwhile, Dimitroglou et al., in their review article, emphasize the utility of transesophageal echocardiogram in the perioperative setting in differing cardiac pathologies.

Beyond mere procedural refinements, several studies within this Issue underscore the identification of individual risk factors in cardiovascular patients. Hu et al., in their retrospective review, identify risk factors associated with functional mitral stenosis following mitral valve repair, which will help guide repair strategies based on patient factors. Akbar et al. describe the role for durable left ventricular assist device implantation and heart transplant with special considerations noted for higher-risk populations, whereas Tracy et al. describe a role for veno-arterial extracorporeal membrane oxygenation in high-risk patients undergoing cardiac catheterization in efforts to bridge a gap in current clinical guidelines. Biancari et al., in their analysis of the European Registry of Type A Aortic Dissection, compare the Bentall and David procedures as durable repair options. These studies reflect a gradual paradigm shift toward increasingly tailored clinical approaches to reduce perioperative morbidity.

This Issue also addresses less commonly encountered but clinically significant disease-specific scenarios and their implications. In a review article by Piscione et al., the authors investigate the lesser-known pathology of carcinoid-induced valve disease, with evolving management strategies including surgical and transcatheter approaches. Barbero et al. pose an alternative to a high-risk approach in redo mitral valve surgeries, by implementing endo-aortic clamping along with retrograde femoral arterial perfusion to avoid injuries associated with surgical re-entry. Fagu et al. present the institutional findings on the impact of chronic total coronary occlusions at the time of surgical revascularization. These contributions further reinforce the movement towards personalized cardiovascular care for patients.

Another important theme explored within this issue is the impact of perioperative risk factors—such as in the single institutional study by Jain et al. which shines a light on the understudied topic of perioperative transient ischemic attacks and their effects on postoperative hospital resource utilization and all-cause readmissions. A comprehensive narrative review by Chandiramani et al. investigates the role of frailty, which is a known predictive factor in postoperative outcomes [4]. They discuss different assessment tools, with an emphasis on the importance of early identification and optimization in vulnerable patients. The topic of pre-revascularization myocardial viability is also explored by Ralota et al., covering current imaging modalities and clinical consensus on their predictive role in patient outcomes.

Beyond perioperative management and operative techniques, Khashkhusha et al. present a literature review of multidisciplinary approaches to deep sternal wound infection, a feared long-term complication following open cardiac surgery. Their review covers treatment methods spanning from medical to surgical management, with descriptive emphasis on reconstructive options in refractory cases. In the realm of proteomic profiling, Zimmermann et al. provide novel insights into potential biomarkers associated with abdominal aortic aneurysm sac behavior after endovascular repair, which may pave the way for better surveillance strategies and therapeutic targeting.

This Special Issue, as a collective of the cardiovascular field ranging from translational science to postoperative outcomes, highlights the efforts by a broad spectrum of cardiovascular specialists to refine and assist with clinical decision making. It is our hope that, by facilitating such multidisciplinary discussion, we can contribute meaningfully to the ever-evolving landscape of cardiovascular disease and the improvement in care for the increasingly complex patient population.
